# Transcriptional and Metabolic Profiling of Potato Plants Expressing a Plastid-Targeted Electron Shuttle Reveal Modulation of Genes Associated to Drought Tolerance by Chloroplast Redox Poise

**DOI:** 10.3390/ijms21197199

**Published:** 2020-09-29

**Authors:** Juan J. Pierella Karlusich, Rocío C. Arce, Fahimeh Shahinnia, Sophia Sonnewald, Uwe Sonnewald, Matias D. Zurbriggen, Mohammad-Reza Hajirezaei, Néstor Carrillo

**Affiliations:** 1 Instituto de Biología Molecular y Celular de Rosario (IBR-UNR/CONICET), Facultad de Ciencias Bioquímicas y Farmacéuticas, Universidad Nacional de Rosario (UNR), Rosario 2000, Argentina; pierella@biologie.ens.fr (J.J.P.K.); arce@ibr-conicet.gov.ar (R.C.A.); 2 Leibniz Institute of Plant Genetics and Crop Plant Research, OT Gatersleben, Corrensstrasse, D-06466 Stadt Seeland, Germany; fshahinnia@gmail.com; 3 Division of Biochemistry, Department of Biology, Friedrich-Alexander-University Erlangen-Nurenberg, 91058 Erlangen, Germany; sophia.sonnewald@fau.de (S.S.); uwe.sonnewald@fau.de (U.S.); 4 Institute of Synthetic Biology and CEPLAS, University of Düsseldorf, Universitätsstr, 1 40225 Düsseldorf, Germany

**Keywords:** drought, stress responses, photosynthesis, chloroplast redox status, flavodoxin, transcriptomics, metabolomics, potato, tuber yield

## Abstract

Water limitation represents the main environmental constraint affecting crop yield worldwide. Photosynthesis is a primary drought target, resulting in over-reduction of the photosynthetic electron transport chain and increased production of reactive oxygen species in plastids. Manipulation of chloroplast electron distribution by introducing alternative electron transport sinks has been shown to increase plant tolerance to multiple environmental challenges including hydric stress, suggesting that a similar strategy could be used to improve drought tolerance in crops. We show herein that the expression of the cyanobacterial electron shuttle flavodoxin in potato chloroplasts protected photosynthetic activities even at a pre-symptomatic stage of drought. Transcriptional and metabolic profiling revealed an attenuated response to the adverse condition in flavodoxin-expressing plants, correlating with their increased stress tolerance. Interestingly, 5–6% of leaf-expressed genes were affected by flavodoxin in the absence of drought, representing pathways modulated by chloroplast redox status during normal growth. About 300 of these genes potentially contribute to stress acclimation as their modulation by flavodoxin proceeds in the same direction as their drought response in wild-type plants. Tuber yield losses under chronic water limitation were mitigated in flavodoxin-expressing plants, indicating that the flavoprotein has the potential to improve major agronomic traits in potato.

## 1. Introduction

Environmental stress represents the most important single factor limiting crop yield worldwide, a situation that will certainly aggravate in the near future as a consequence of global climate change [1,2]. Among the adverse conditions that constrain plant growth and reproduction, drought has the highest impact in quantitative terms [3]. Water restriction negatively affects photosynthetic rates by decreasing CO_2_ availability as a result of stomatal closure, and by feedback inhibition due to limitations in photosynthate transport to sink organs [4,5]. Many changes associated to water deficit are therefore detected in the leaves and, accordingly, drought responses are inextricably linked to photosynthesis and chloroplast biochemistry [6].

Potato is the third most important food crop in the world [3,7], and is vulnerable to drought, salinity and other environmental stresses, which affect tuber yield and quality [8,9]. The situation is particularly critical in developing countries where potato is most important as an affordable and nutritionally rich food supply [5], and where the impact of global climate change is predicted to be more severe [1,9]. Therefore, breeders face increasing pressure to develop new lines with improved drought tolerance while keeping high crop yield, tuber quality and market acceptance [5].

Water limitation elicits a very complex plant response, which combines physiological, cellular and metabolic adaptations to the stress situation, and involves genome-wide changes in gene expression patterns. While many drought-responsive genes have been identified, it is presently difficult to define the role played by most of them in the tolerance against this environmental challenge [10]. As many other abiotic stresses, water deficit causes a rise of reactive oxygen species (ROS) levels, especially in leaves [11]. Indeed, Vasquez-Robinet et al. [8] reported that the higher drought tolerance displayed by Andean potato genotypes was related to enhanced expression of genes encoding antioxidant proteins located in chloroplasts. Moreover, potato transformation with genes related to ROS scavenging led to lines exhibiting improved performance under water deprivation [12,13,14], indicating that manipulation of ROS metabolism is a promising strategy to improve drought tolerance [11].

We have generated tobacco plants with increased tolerance to multiple sources of abiotic stress by introducing a cyanobacterial flavodoxin (Fld) directed to chloroplasts [15,16]. Fld is an electron carrier flavoprotein present in bacteria and some algae that displays essentially the same activities and redox interactions as the iron-sulfur protein ferredoxin (Fd), including functional integration as a final electron acceptor in the photosynthetic electron transport chain (PETC). Fld expression is normally induced in microorganisms under conditions of iron starvation and environmental stress that cause Fd down-regulation, taking over Fd functions as its levels decline, and preventing over-reduction of the PETC and ROS propagation [16,17]. Long before the advent of terrestrial plants, the Fld gene disappeared during the evolutionary transition of the green algal lineage from the open ocean to freshwater, correlating with changes in iron bioavailability across habitats (low in the ocean, high in freshwater) that impact Fd levels [18]. However, introduction of a plastid-targeted Fld in various plant species improved delivery of reducing equivalents to productive pathways of the chloroplast, which in turn restricted plastid ROS production and increased tolerance to drought and other stresses [15,16,19,20,21,22].

The need for drought-tolerant potato lines prompted us to evaluate the Fld approach in this crop. We describe herein the preparation and characterization of potato plants expressing a plastid-localized Fld, and displaying improved photosynthesis, growth and tuber yield under conditions of water deprivation. To gain further insights into the mechanism(s) of Fld-associated stress tolerance, we generated genome-wide transcript profiles from wild-type (WT) and Fld-expressing leaves at a pre-symptomatic stage of water restriction, and combined this approach with metabolic profiling of carbohydrates and amino acids. The results provide a detailed snapshot of how chloroplast redox biochemistry affects gene expression, metabolism and yield in a major crop during this agronomically relevant abiotic stress.

## 2. Results

### 2.1. Expression of Plastid-Targeted Fld Improved Potato Drought Tolerance

Potato plants were transformed with the *fld*-containing plasmid described by Tognetti et al. [15] using published methods ([23]; Figure S1a; see Materials and Methods). Several independent *Stpfld* lines (for ***S****olanum **t**uberosum* **p**lastidic **Fld**) were obtained, expressing different Fld levels as revealed by sodium dodecyl sulfate–polyacrylamide gel electrophoresis (SDS-PAGE) and immunoblotting (Figure S1b). The flavoprotein was recovered from leaf extracts as a mature-sized product (Figure S1b), suggesting plastid import and processing [15]. Traces of Fld precursor and processing intermediates were detected in highly expressing *Stpfld252* and *Stpfld239* lines (Figure S1b), as already observed in tobacco [15].

**Figure 1:**
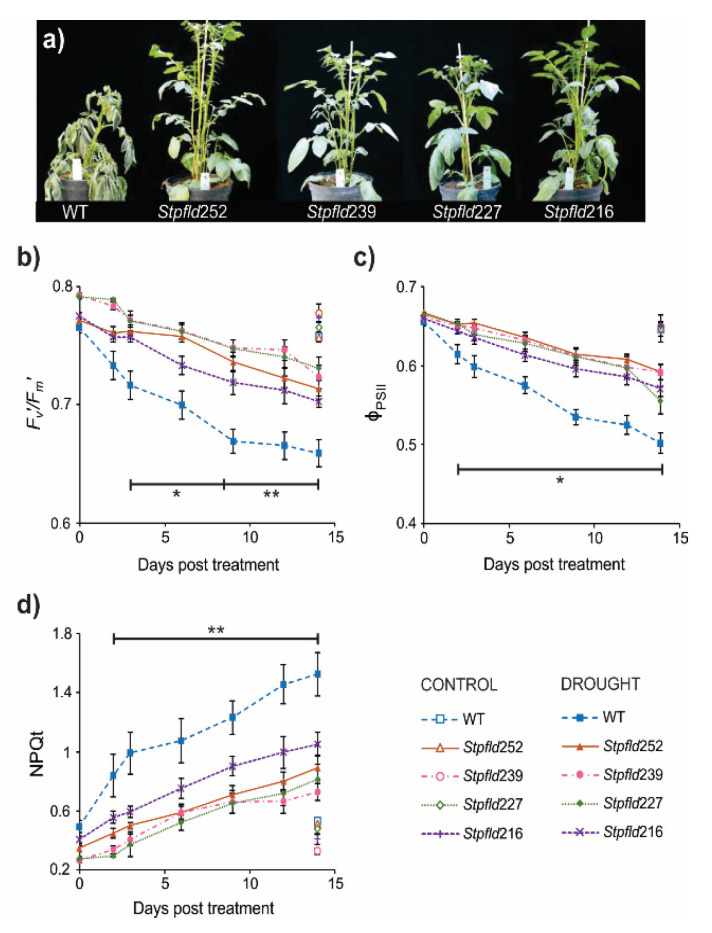
Phenotypes and photosynthetic activities of wild-type (WT) and Fld-expressing potato plants under short-term drought treatment. Thirty-days old plants were exposed to hydric stress under growth chamber conditions by interrupting irrigation. (**a**) Plants were photographed after 14 days of water withdrawal. (**b**–**d**) Photosynthetic parameters *F*’_v_/*F*’_m_ (**b**), ϕ_PSII_ (**c**) and NPQt (**d**) were determined at the indicated days of treatment, as described under Materials and Methods. Values are means ± SE of 4–6 biological replicates, and asterisks indicate statistically significant differences with the wild type using ANOVA and Tukey’s Multiple Comparison Test at *p* ≤ 0.1 (*) or *p* ≤ 0.05 (**).

To evaluate the drought tolerance conferred by Fld introduction, 30-days old WT and *Stpfld* plants cultured under growth chamber conditions (see Materials and Methods) were exposed to water deficit by interrupting irrigation. Visual symptoms of stress were observed in WT leaves after ~9 days of treatment, and by 14 days wilting extended to both leaves and stems (Figure 1a), correlating with significant decreases in leaf relative water contents (RWC, Figure S2). Under the same conditions, Fld-expressing plants looked healthy (Figure 1a) and retained leaf turgor (Figure S2).

As indicated, photosynthesis is a most sensitive target of water deficit. Measurements of chlorophyll *a* fluorescence on WT leaves revealed a fast decrease in the *F*_v_’/*F*_m_’ ratio after only 2–3 days of stress (Figure 1b). This parameter is customarily used to monitor damage to photosystem (PS) II [24]. The quantum yield of PSII (Φ_PSII_), which provides an estimation of electron flow through this photosystem, also declined steadily with the days of treatment (Figure 1c). Finally, dissipation of the excess of energy not used for photochemistry, a process monitored by the non-photochemical quenching of chlorophyll fluorescence (NPQt), increased as stress became more severe (Figure 1d). The results indicate that drought-associated photosynthetic impairment preceded visible tissue dehydration and increased as wilting progressed (Figure 1b–d). These detrimental effects were largely prevented by Fld presence in chloroplasts of the transgenic plants, as indicated by differential preservation of PSII integrity and electron flow, and comparatively lower values of NPQt (Figure 1b–d). Only minor differences, without statistical significance, were observed in well-watered plants of all lines during the timespan of the assay (Figure 1b–d).

**Figure 2:**
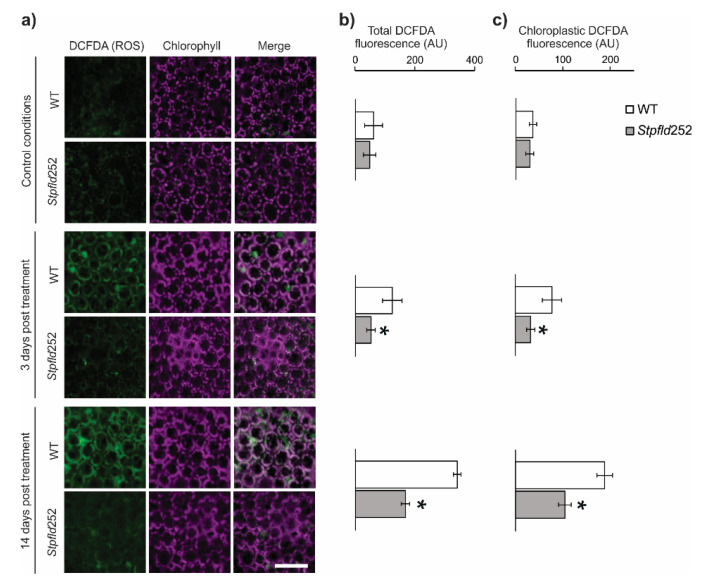
ROS accumulation in WT and Fld-expressing potato leaves under short-term drought treatment. ROS were visualized by fluorescence microscopy after leaf infiltration with 2′,7′-dichlorodihydrofluorescein diacetate (DCFDA). (**a**) Confocal microscopy analysis of subcellular ROS accumulation in leaves of 30-days old WT and *Stpfld252* plants after 0, 3 and 14 days of water withdrawal. Images show ROS fluorescence (left, green), chlorophyll auto-fluorescence (middle, magenta), and the merge of the two channels (right). Scale bar: 40 μm. Quantification of ROS levels in whole-leaf tissue (**b**) and chloroplasts (**c**) of WT and *Stpfld252* plants. Results are means ± SE of 5 replicates, and asterisks indicate statistically significant differences between lines using ANOVA and Tukey’s Multiple Comparison Test (* *p* ≤ 0.05).

ROS build-up is also a common feature of environmental stress conditions [11,25], and the protective effect of Fld has been linked to its role as a general antioxidant specific for chloroplasts [16,17,26]. ROS accumulation was visualized by confocal laser scanning microscopy (CLSM) in leaves of water-deprived plants infiltrated with 2′,7′-dichlorodihydrofluorescein diacetate (DCFDA). Results are illustrated in Figure 2 for *Stpfld252* plants, a line displaying high levels of Fld expression (Figure S1b) and drought tolerance (Figure 1). Under the conditions employed, DCFDA fluorescence was nearly undetectable in watered plants from both genotypes (Figure 2a). At 14 days of water withdrawal, WT leaves showed significant increases in ROS-associated fluorescence (Figure 2b). Actually, ROS build-up was already evident after only 3 days of treatment, indicating that increased production of these reactive species was an early manifestation of the plant stress response (Figure 2b). Presence of plastid Fld largely prevented this rise (Figure 2b). About 60% of total ROS was associated to chloroplasts in both conditions and genotypes (Figure 2c).

### 2.2. Microarray Analysis of Drought-Stressed Plants Expressing Chloroplast Fld

Most transcriptome analyses on water-restricted potatoes have been performed on plants showing stress symptoms [27,28,29]. However, Fld provided protection against drought effects occurring at a pre-symptomatic stage that might contribute to the differential tolerance exhibited by Fld-expressing plants. Therefore, a genome-wide transcriptional profiling was carried out using WT and *Stpfld252* leaves sampled at 3 days of treatment as RNA source, together with their corresponding watered controls. Once again, the *Stpfld252* line was chosen because of its high levels of Fld accumulation and drought protection.

Gene expression profiles were determined using a single-channel Agilent potato microarray consisting of 42,034 probes. We did not carry out any filtering for potentially redundant probes mapping the same transcript due to the difficulties related to the highly heterozygous nature of potato genomes, their polyploidy variance, and the fact that different accessions were used for the microarray design, the reference genome sequencing and our current work. Functional annotation was based on the Mapman ontology using the GoMapman website resource [30]. More than 80% of the genes included in the microarray could be assigned to a functional category. The total number of leaf-expressed genes that passed the background correction and filtering processes was similar for the two genotypes under control conditions and represented about 75% of the total genes present in the microarray.

### 2.3. Chloroplast Fld Affected Gene Expression Patterns of Potato Leaves in the Absence of Stress

**Figure 3:**
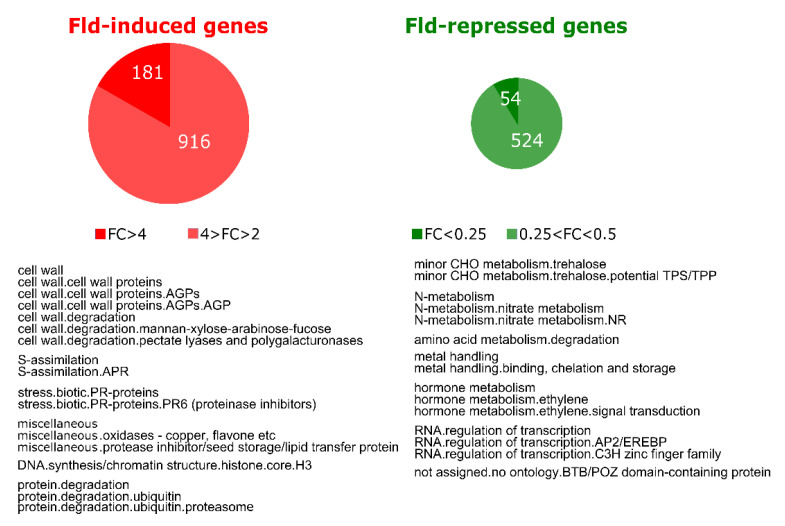
Effect of Fld expression on the potato transcriptome. Upper part: pie charts showing the number of genes that were induced (FC > 2 and FDR < 0.05) or repressed (FC < 0.5 and FDR < 0.05) by Fld in leaves of 30-days old *Stpfld252* plants under growth chamber conditions. Lower part: over-representation analysis of Mapman functional categories among Fld-responsive genes. The analysis was carried out separately for induced and repressed genes (Fisher’s exact test with Bonferroni correction and FDR < 0.05). The list of genes and their corresponding descriptions, functional assignments and FC values are described in Table S1.

We first evaluated the effect of Fld on gene expression in leaves of potato plants grown under normal conditions. Analysis of transcript levels in WT and *Stpfld252* leaves showed that 1097 genes were induced at least 2-fold by Fld relative to WT siblings, with 181 increasing more than 4-fold. Moreover, 578 transcripts declined to 50% or less in *Stpfld252* leaves compared to the WT, 54 of which accumulated below 25% (Figure 3, Table S1). Among genes induced in *Stpfld252* plants there was a remarkable over-representation of those associated to protein degradation by the proteasome (Figure 3, Table S1). A similar induction pattern has been observed in Fld-expressing tobacco plants [31], indicating that plastid-located Fld exerts a key regulatory role in the accumulation of proteasomal components in different species, presumably through modulation of chloroplast redox chemistry.

Functional categories that displayed differential repression by Fld comprised trehalose synthesis, nitrate metabolism, amino acid degradation and genes associated to metal uptake and utilization such as Cd- and Al-induced proteins, Cu chaperones and Fe-chelate reductases. Noteworthy, ethylene metabolism and signaling were consistently down-regulated in *Stpfld252* plants, including 29 genes encoding transcription factors of the apetala2/ethylene-responsive family (Figure 3, Table S1). These observations also agree with earlier reports on Fld-expressing tobacco plants [31].

### 2.4. Hydric Stress Caused Extensive Transcriptional Reprogramming in Both WT and Fld-Expressing Leaves

**Figure 4:**
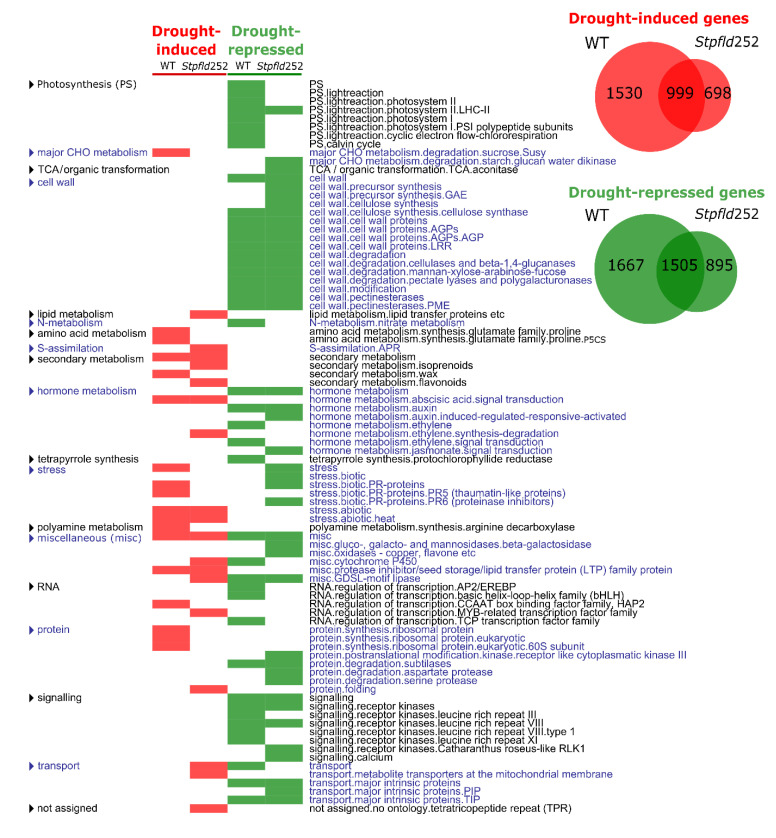
Drought stress led to extensive transcriptional reprogramming in both WT and Fld-expressing plants. Venn diagrams of genes differentially expressed (DE) in response to drought are shown in the right upper corner. Genes were defined as induced when FC > 2 and FDR < 0.05, and as repressed when FC < 0.5 and FDR < 0.05. On the left, list of functional categories with over-represented DE genes during the response of WT and *Stpfld252* plants to drought. The analysis was carried out separately for induced and repressed genes (Fisher’s exact test with Bonferroni correction and FDR < 0.05). The list of genes and their corresponding descriptions, functional assignments and FC values are described in Table S2.

Distribution of differentially expressed (DE) transcripts between treatments and genotypes (Venn diagrams in Figure 4) indicates that 999 genes were induced by drought in both lines, 1530 genes only in the non-transformed plants, and 698 uniquely in *Stpfld252* leaves, in all cases with a fold-change (FC) > 2 and a false discovery rate (FDR) < 0.05 (see Materials and Methods for details). In contrast, 1505 genes were repressed by drought in both lines, 1667 only in the WT and 895 exclusively in *Stpfld252* plants. Hence, Fld mitigated changes in gene expression (induction or repression) driven by the drought treatment.

Functional enrichment analysis identified functional categories significantly affected by water limitation in the two genotypes. Among the categories that showed drought-dependent induction in both WT and *Stpfld252* plants, abiotic stress was over-represented. The opposite situation, e.g., pathways repressed by drought in the two lines, is exemplified by cell wall metabolism and kinase-dependent protein modification and signaling (Figure 4, Table S2).

Several DE genes showed differential stress responses in only one genotype. Drought-dependent inactivation of photosynthesis in WT plants (Figure 1b–d) was paralleled at the transcript level by widespread down-regulation of genes encoding photosynthetic components. Repression particularly impaired light reactions, and was largely prevented by chloroplast Fld (Figure 4, Table S2), correlating with the protection of photosynthetic activities in the transformant.

While the effect of drought on leaf gene expression was predominantly repressive, stress-dependent induction was also important in WT plants with more than 2500 genes affected. Enriched functional categories included protein metabolism with 98 DE genes (mostly ribosomal proteins), biotic stress and amino acid synthesis (Figure 4, Table S2). The differential induction of these pathways was partially abolished by Fld expression in chloroplasts, suggesting once again that water deficit had less impact in the transgenic plants than in their WT siblings.

Finally, some functional categories were specifically over-represented in the transformant, including drought-associated up-regulation of membrane transport, sulfur assimilation and flavonoid synthesis (Figure 4, Table S2). Accumulation of leaf flavonoids has been correlated with enhanced tolerance to both oxidative and drought stresses in Arabidopsis [32].

### 2.5. Expression of One-Third of Drought-Responsive Genes Was Not Affected by Fld

Clustering of DE transcripts allows identification of gene groups that display similar expression patterns and presumably share common regulatory pathways. Leaf transcripts detected in our microarray were thus grouped into 47 clusters of widely different size. The more populated (27,622 members, ~85% of all leaf-expressed genes) included transcripts whose levels were not affected by treatment or genotype. Of the remaining 46 clusters containing genes differentially expressed in response to water limitation and/or Fld presence (4847 genes), we focused in 13 clusters that were either highly populated or enriched in pathways and functional categories associated to stress responses, redox biochemistry and chloroplast functions, comparing *vis-à-vis* those that exhibited contrasting expression behaviors (e.g., induction vs. repression). DE transcripts contained in these clusters can be found in Tables S4–S6, while the other 33 clusters are described in Figure S3.

Genes that display a similar drought response in the presence or absence of Fld represent 31.5% of total DE transcripts, of which 601 were induced (cluster 1) and 929 repressed (cluster 2). Cluster 1 was enriched in genes coding for components of the cytosolic branch of glycolysis, ethylene synthesis, heat stress and signal transduction associated to abscisic acid (ABA), whereas cluster 2 included genes belonging to cell wall metabolism such as cellulose synthases, cellulases and pectinesterases (Figure S4; Table S3). Several receptor kinases of the leucine-rich repeat family were also repressed by drought irrespective of genotype.

### 2.6. Genes Whose Drought Response Was Ameliorated by Chloroplast Fld

**Figure 5:**
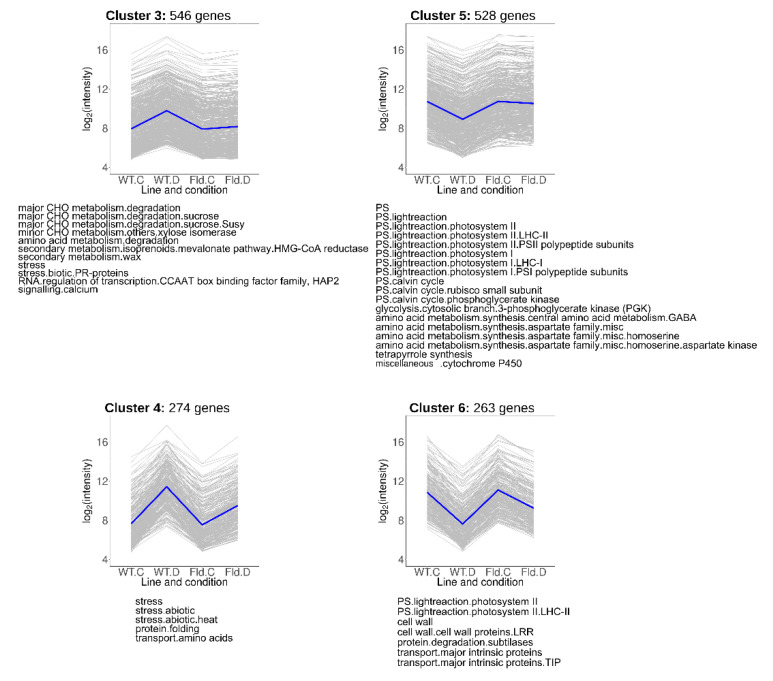
Clusters containing genes in which the drought response was abolished or ameliorated by Fld expression. Each gray line of the charts corresponds to a particular gene, and the dark line represents the average behavior of all the genes contained in each cluster. Labels in the abscissa correspond to the WT line under control (WT.C) and drought conditions (WT.D), and the *Stpfld252* line under control (Fld.C) and drought conditions (Fld.D); those in the ordinates correspond to the FC values in log_2_ scale. For each pairwise comparison between lines or treatments, genes were defined as induced when FC > 2 and FDR < 0.05, and repressed when FC < 0.5 and FDR < 0.05. The total number of genes in each cluster is indicated above the corresponding panel, and the over-represented functional categories are shown below (analyzed using Fisher’s exact test with Bonferroni correction and FDR < 0.05). The list of genes belonging to these clusters and their corresponding descriptions, functional assignments and FC values in the multiple comparison tests are described in Table S4.

Transcripts that showed lower induction (Clusters 3 and 4) or repression (Clusters 5 and 6) in *Stpfld252* leaves relative to WT counterparts under water stress conditions comprise 33% of total DE genes (1611), and collectively represent the most common response to treatment and genotype (Figure 5, Table S4). Cluster 3 contained 546 genes whose drought-dependent induction was completely abolished by Fld, while for 274 genes of Cluster 4 the effect of Fld was only partial.

Cluster 3 contained genes associated to sucrose metabolism and isoprenoid synthesis (Figure 5, Table S4). Isoprenoids are a large and diverse group of molecules that include photosynthetic pigments (chlorophylls, carotenoids), components of different electron transport chains (plastoquinone, ubiquinone), hormones (ABA, gibberellins, cytokinins and brassinosteroids), membrane-associated phytosterols and antimicrobials (phytoalexins). Cluster 4 was enriched in pathways related to abiotic stress responses, including various heat-responsive transcription factors and heat-shock proteins (Figure 5, Table S4).

Clusters 5 and 6 were enriched in genes associated to photosynthesis, including components of the light-harvesting complexes, the PETC and the Calvin-Benson cycle (Figure 5, Table S4). In addition, Cluster 5 (528 genes) includes transcripts encoding enzymes involved in glycolysis and in homoserine and γ-aminobutyrate (GABA) synthesis. Cluster 6 (263 genes), in turn, contained transcripts related to cell wall metabolism, although most of the genes belonging to this functional category were grouped in Cluster 2.

### 2.7. Fld-Induced Genes That Were Repressed by Water Limitation

**Figure 6:**
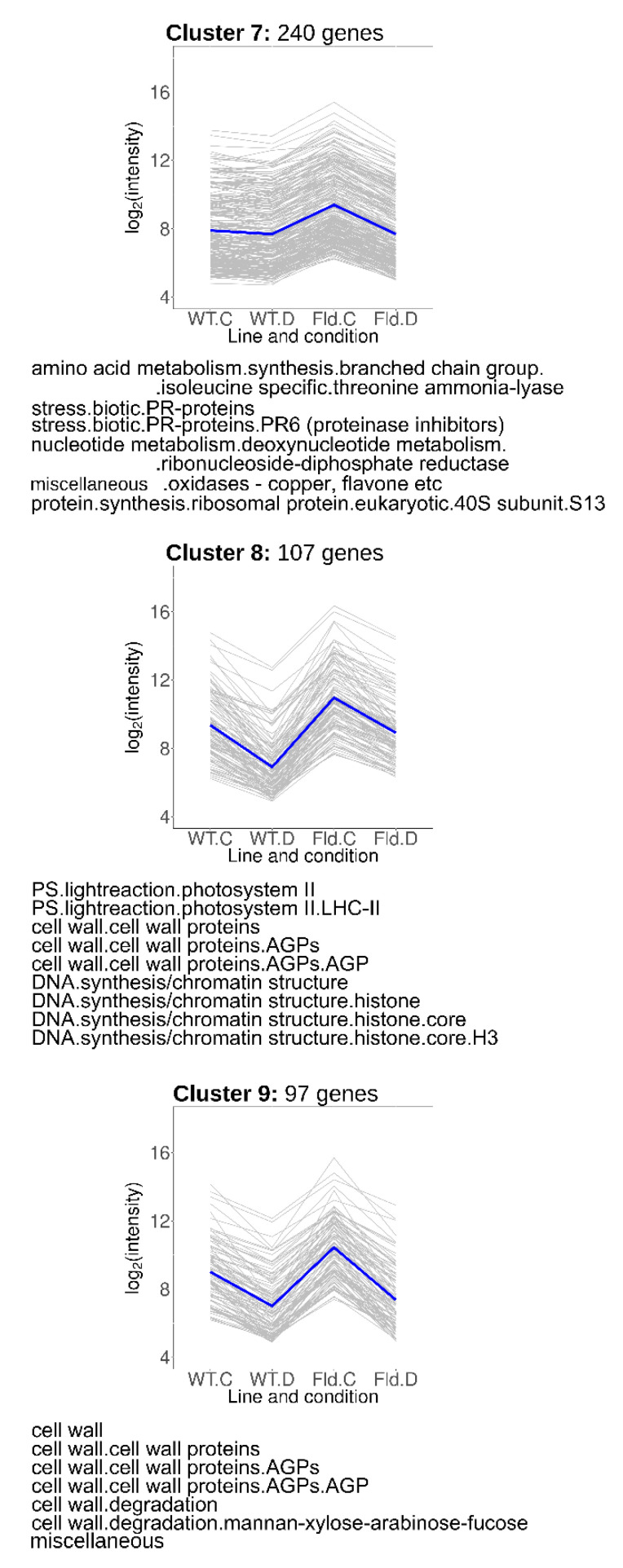
Genes regulated by Fld and drought in opposite directions. Each gray line of the charts corresponds to a particular gene, and the dark line represents the average behavior of all cluster genes. Labels of the abscissa and ordinates, total number of genes in each cluster and list of over-represented pathways are indicated as in Figure 5. Further details are provided in Table S5.

Genes grouped in Clusters 7–9 (Figure 6) were induced under control conditions in *Stpfld252* plants, accompanied by drought-related repression in the transformant (Cluster 7), or in both lines (Clusters 8 and 9). Cluster 7 (240 members) included genes associated to biotic stress, amino acid synthesis and protein and nucleotide metabolism (Figure 6, Table S5). Cluster 8 was enriched in photosynthetic genes, particularly components of PSII and its light-harvesting antenna, and cell wall proteins. Finally, cluster 9 included fasciclin-like arabinogalactan proteins, which were reported to participate in fiber initiation and elongation, and to contribute to the integrity of the primary cell wall matrix [33]. DE transcripts belonging to the cell wall functional category were also shared with Clusters 2 and 6.

### 2.8. Drought-Responsive Genes Primed by Fld Expression

**Figure 7:**
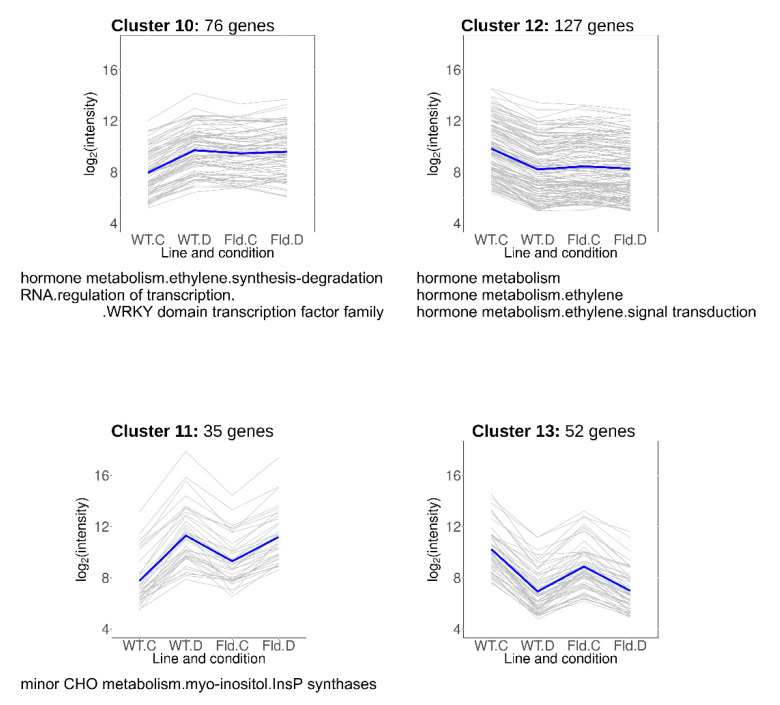
Drought-responsive genes primed by Fld in the absence of stress. Each gray line of the charts corresponds to a particular gene, and the dark line represents the average behavior of all cluster genes. Labels of the abscissa and ordinates, total number of genes in each cluster and list of over-represented pathways are indicated as in Figure 5. Further details are provided in Table S6.

Clusters 10–13 included DE transcripts that responded to water limitation but were also modulated by Fld in the same direction under normal growth conditions (Figure 7, Table S6). Cluster 10 contained 76 transcripts induced by chloroplast Fld to the levels attained in drought-stressed WT plants, while genes grouped in cluster 11 (35 members) were further up-regulated by water limitation in both WT and *Stpfld252* plants. DE genes over-represented in these clusters belonged to the ethylene signal transduction and minor carbohydrate functional categories, including a myo-inositol-1-phosphate synthase (MIPS) that catalyzes the first step of myo-inositol biosynthesis.

Cluster 12 comprised 127 genes whose expression was repressed by Fld under growth chamber conditions to the levels attained by the drought treatment in the two lines (Figure 7, Table S6). Most conspicuous among them was the ethylene responsive factor 5. Finally, cluster 13 included 52 transcripts that were repressed by Fld in the absence of stress, and further by drought in both lines (Figure 7, Table S6), with no specifically enriched functional category.

### 2.9. Comparative Expression of Selected DE Transcripts in WT and Stpfld252 Leaves

Expression of several DE genes was evaluated by quantitative reverse-transcription (qRT)-PCR to validate the results obtained with the microarray experiments. Genes were selected on the basis of their stress responses. Out of 10 DE genes assayed, 4 showed a strict correlation between the microarray and qRT-PCR data, including the FC (Figure S5). They belonged to clusters 14 (proteasomal RPN9b) and 5 (*psaK*, starch synthase GBSSI and nitrate reductase NR3). Other 5 genes displayed quantitative differences in FC between the two procedures that did not modify cluster assignment (Figure S5). Clusters 2 (GAST), 5 (*psbY*, Fd1), 11 (transcription factor ATHB7) and 14 (aminocyclopropane-1-carboxylate oxidase ACO1) were represented in this group. Finally, another component of the proteasomal system, PSα2β, would be allocated to cluster 34 on the basis of qRT-PCR (Figure S5), and to cluster 14 by the microarray analysis (Figure S3). Noteworthy, the change in cluster assignment was caused by a single significant difference in the FC obtained for stressed WT plants (Figure S5).

### 2.10. Fld Presence Prevented the Increase in Amino Acid Levels in Leaves of Drought-Exposed Plants

**Figure 8:**
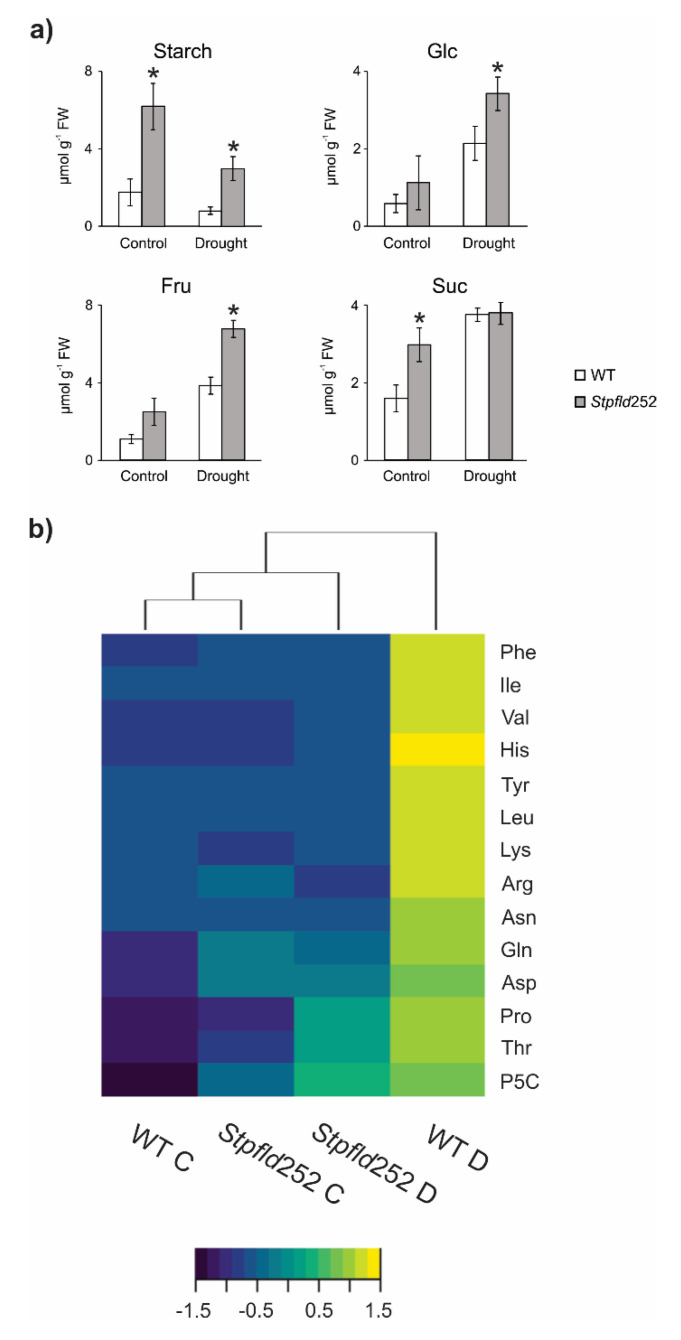
Effect of chloroplast Fld on carbohydrate and amino acid levels under water limitation. Extracts were prepared from leaves of 30-days old plants at 3 days of water deprivation and from their watered controls (8 h within the light period), and the levels of the indicated sugars and amino acids were determined as described under Materials and Methods. (**a**) Carbohydrate contents are given as means ± SE of 5–8 independent plants. Statistically significant differences between lines are shown by asterisks and were determined using ANOVA and Tukey’s Multiple Comparison Test (* *p* ≤ 0.05). FW, fresh weight. (**b**) Heat map of amino acids assayed for the different lines and treatments. Color scale corresponds to the standardized scores (dark blue for low values, yellow for high values). Quantitative data of amino acid determinations are shown in Figure S6. C, control conditions; D, drought. Heat maps were produced in R language using the heatmap. 2 function of the gplots package.

When plants were grown in the absence of stress, contents of starch and the soluble carbohydrates glucose, fructose and sucrose were higher in *Stpfld252* leaves compared to WT siblings (Figure 8a). Water limitation, in turn, led to a major decline in starch in both WT and *Stpfld252* leaves, with concomitant increases in soluble sugars (Figure 8a).

Unlike carbohydrates, most amino acids accumulated to similar levels in leaves from the two genotypes grown under normal conditions, with the conspicuous exception of Pro and its related metabolite pyrroline-5-carboxylate (P5C), whose levels were significantly higher in the transformant (Figure S6). Ala also showed moderately increased contents in *Stpfld252* leaves (Figure S6).

With few exceptions (Met, Ala, Glu, GABA and aminocyclopropane-1-carboxylate), amino acid contents were up-regulated by water deprivation in WT leaves. These drought-dependent increases were completely prevented by chloroplast Fld for 11 proteinogenic amino acids and ameliorated in three more (Pro, P5C and Thr), whereas Ser and Gly build-up was not affected (Figure 8b, Figure S6). As expected, drought caused a major increase in the stress marker proline and its precursor P5C, which can act as compatible osmolytes and antioxidants [34]. The lower levels observed in *Stpfld252* leaves (Figure 8b, Figure S6) correlated with their increased stress tolerance.

### 2.11. Fld Improved Growth and Tuber Yield under Chronic Water Restriction

Impact of drought on tuberization depends not only on the stress intensity but also upon timing, with the most damaging effects on yield occurring when the stress condition was applied at the stolon and tuber initiation stages [35]. Tuber yield was thus determined to evaluate possible Fld effects during a long-term water restriction regime with episodic rehydration. WT and *Stpfld252* plants were grown in soil at 100% field capacity (FiC) for 30 days, at the time when stolons were set. Water irrigation was interrupted until soil reached 40% FiC, rehydrated to 70% FiC and this protocol repeated for a total treatment of 90 days (Figure S7), when tuberization was extensive in both WT and *Stpfld252* lines. The protocol applied was similar to those employed to compare potato genotypes with different drought susceptibilities [36].

**Figure 9:**
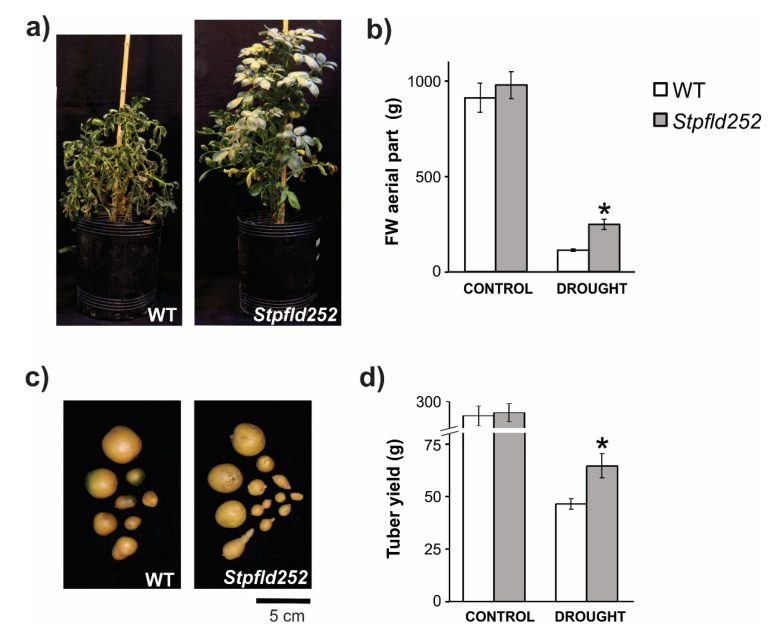
Phenotypes of WT and Fld-expressing potato plants under long-term drought treatment. Plants were grown in soil for 30 days at 100% FiC. Water irrigation was then interrupted until soil reached 40% FiC, rehydrated to 70% FiC and this protocol repeated for a total treatment of 90 days (Figure S7). (**a**) Plants were photographed after 90 days of drought treatment. (**b**) Fresh weight of aerial parts. (**c**) Representative photograph of tubers. (**d**) Tuber yield. Results are means ± SE of 5 replicate plants, and asterisks indicate statistically significant differences between lines using non-parametric Mann–Whitney test (* *p* ≤ 0.05).

Water restriction affected aerial growth in both lines, but significantly less in the transformant. Compared to control watered conditions, stressed WT plants accumulated only 10% aerial fresh weight (FW) at the end of the 90-days treatment, whereas *Stpfld252* siblings reached ~25% (Figure 9a,b). Differential stress protection by Fld was also reflected at the level of tuber production. Water limitation reduced tuber yield by more than 80% in WT plants, but less than 70% in their *Stpfld252* counterparts (Figure 9c,d), resulting in absolute yields of 65 ± 6 vs. 48 ± 2 g per plant for *Stpfld252* and WT lines, respectively (Figure 9d).

## 3. Discussion

Drought is one of the major abiotic stresses affecting agronomic productivity worldwide. Water restriction exerts its negative effect at various levels, and the plant protective responses exhibit a comparable complexity. Stomatal closure is among the earliest, aimed at preventing water loss through transpiration [37]. An unwanted consequence of this defensive mechanism is the inhibition of gas exchange and CO_2_ assimilation, which in turn leads to NADPH build-up and down-regulation of photosynthetic electron transport due to limitation of electron acceptors (oxidized Fd and NADP^+^). The excess of reducing equivalents accumulated in the PETC might increase adventitious O_2_ reduction and ROS propagation, triggering redox-based signaling pathways and eventually oxidative damage [11]. Plant responses to this particular aspect of the drought syndrome include a suite of alternative electron transport pathways that dissipate the surplus of excitation energy from the PETC and/or export reducing equivalents to other cellular compartments. They comprise cyclic electron transport, photorespiration, chlororespiration, flavin-diiron proteins, the malate valve and the Mehler-Asada cycle [11]. However, drought also causes down-regulation of many photosynthetic components including Fd [27,28,38]. While this response alleviates the hazardous combination of light absorption and highly reduced redox intermediates in a context of elevated oxygen levels, the final outcome is further inactivation of photosynthesis.

As indicated, Fld redox properties largely match those of Fd, and the functional substitution of Fd by Fld allows algae and cyanobacteria to survive and reproduce in hostile environments [17]. Indeed, the flavoprotein is typically induced as an adaptive resource under environmental hardships that compromise Fd expression and activity, including osmotic stress, high light, heat, oxidants and especially, iron starvation (reviewed in [17,39]). Moreover, the introduction of Fld in plants via genetic engineering increased tolerance to multiple stresses [15,22,40] and complemented Fd-deficient plants [41]. While this protective effect is clearly associated to Fd substitution, Fld affects plant development [22,42], and transcriptional profiles (Figure 3, [31]) in the absence of stress, when Fd levels are not down-regulated. The functional basis for these effects remain yet to be elucidated, but they depend on the relative proportions of the two electron carriers [40], suggesting that they interact with common partners in the redox network of the chloroplast, and that the magnitude of the Fld impact might vary between species accumulating different Fd amounts. Analysis of plastoquinone (PQ) redox status revealed that Fld prevented over-reduction of the PETC [40], indicating that the flavoprotein was able to function as electron sink in chloroplasts despite eons of evolutionary divergence between cyanobacteria and terrestrial plants [18]. These promising results encouraged application of the Fld approach to crops and to the most agronomically relevant stress, drought.

We showed herein that early drought effects on photosynthetic activity and ROS build-up were protected by chloroplast Fld (Figure 1 and Figure 2). In spite of this protection, and of previous reports showing induction of various antioxidants under adverse environmental conditions [12,43,44], antioxidant metabolism was not over-represented as a functional category in any cluster. While ROS levels were already increased in WT leaves by the time of RNA collection, the antioxidant defense systems were not significantly induced at this early stage of the drought treatment (Table S7). Moreover, several ROS scavenging enzymes and proteins were actually down-regulated, and the protection afforded by Fld against this repression (Table S7) might critically contribute to the drought tolerance and lower ROS build-up displayed by the transformants.

After 3 days of water deprivation, 5701 transcripts were differentially expressed in WT plants, but only 4097 in Fld-expressing siblings (Figure 4). Then, the main effect of Fld presence in quantitative terms was to mitigate the changes in gene expression driven by the drought treatment, displaying an attenuated response that affected many functional categories (Figure S8).

The down-regulation of genes encoding photosynthetic components is an universal feature of water limitation [27,28,29,38], which aggravates the direct inhibition of photosynthesis through stomatal closure and acceptor side limitation. Under our conditions, 3 days of treatment were sufficient to cause significant repression of transcripts coding for members of the light-harvesting complexes, the PETC and the Calvin-Benson cycle (Figure 4, Figure 5 and Figure 6), even though the decrease in photosynthetic activity was only moderate at this stage (Figure 1b), presumably reflecting slow turnover of the corresponding proteins.

Fld expression provided partial or complete protection against this repression. It is worth noting that photosynthesis was over-represented as a functional category in Clusters 5, 6 and 8 (Figure 5 and Figure 6), but not in cluster 2, which harbored those genes whose drought-dependent repression was not affected by Fld presence (Figure S4), underscoring the relevance of the flavoprotein for the preservation of photosynthetic activity in the stressed plants.

A most conspicuous functional category among induced genes was stress, enriched in Clusters 1, 3, 4 and 7 (Figure 5 and Figure 6 and Figure S4), indicating that Fld mitigated the plant stress response in most cases. While abiotic stress concentrated specifically in clusters 1 and 4, biotic stress was over-represented in clusters 3 and 7, including transcripts encoding pathogenesis-related (PR) proteins. Members of the PR-6, PR-2 (endo-β-1,3-glucanases) and PR-12 (defensins) families were present in Cluster 7, and chitinases in cluster 3.

Genes found in clusters 10–13 responded to Fld presence under control conditions in the same direction as they did in the WT under drought (Figure 7). We coined the term priming to describe genetic traits displaying this behavior [31]. Priming might help the plant to better cope with the environmental challenge, thus representing a cause of the increased drought tolerance. Indeed, priming has been reported to substantially contribute to the phenotypes of drought-tolerant barley varieties, which exhibited stressed-like expression patterns in the absence of stress [45]. Analogous to our observations, drought caused stronger transcriptional changes in sensitive barley genotypes compared to tolerant varieties [45,46,47]. Enriched functional categories among primed potato genes included induction of myo-inositol synthesis (Figure 7). Overexpression of a MIPS-encoding gene enhanced inositol levels and salt stress tolerance in Arabidopsis, tobacco and rice [48,49,50].

The effects of drought on cell wall metabolism and architecture are complex and depend on the plant species, genotype and age [51]. Under the conditions employed here, the overall effect of water limitation was repressive, with only marginal protection by Fld (Figure 4). Cluster analysis, in turn, confirmed that most functional categories related to cell wall metabolism grouped in Cluster 2 (Figure S4), although they were also found in Clusters 6, 8 and 9 (Figure 5 and Figure 6). Inclusion in these clusters implied that the relevant genes were down-regulated by drought in the WT, the transformant or both. Genes encoding xyloglucan endotransglucosylases/hydrolases, pectin methylesterases and expansins were extensively represented in these clusters (Tables S4–S6), indicating that both matrix properties and hemicellulose deposition were compromised by the drought treatment [51,52]. The results also suggest that the stress tolerance conferred by Fld expression was not related to the protection of cell wall metabolism.

In previous transcriptomic studies on potato and other crops, RNA was usually collected at stages of the treatments in which plants already exhibited stress symptoms [27,28,29]. Comparison of the functional categories over-represented among DE genes in those and our studies revealed many similarities such as repression of photosynthetic genes [27,28]. In addition, stress-repressed genes involved in cell wall metabolism in our transcriptional profiling were the same as those reported by Evers et al. [27], and some genes involved in abiotic stress such as heat-shock proteins were also shared in several studies [27,29]. The results suggest that modulation of these stress-responsive genes was initiated early and maintained as the adverse condition proceeds. Direct comparison of early vs. late drought responses in Arabidopsis further supports this contention [53].

Phenotypic effects exerted by chloroplast-located Fld were also evident in the levels of leaf carbohydrates and amino acids. Increased accumulation of transient starch in the absence of stress might result from the higher photosynthetic activity displayed by Fld-expressing leaves [15,26,54]; its preservation under drought from a lower repression of photosynthetic genes (Figure 5 and Figure 6), including starch synthase (Figure S5, Table S4). Water limitation led to a strong increase in amino acid levels, most conspicuously Pro, in WT leaves, an effect that was significantly attenuated by Fld presence (Figure 8b, Figure S6). A good correlation was found between induced amino acid levels and some genes responsible for their synthesis, such as those associated to Pro, Asp, Thr, Tyr and branched chain amino acids metabolism (Table S1, Figure 8b). Up-regulation of nitrogen mobilization has been associated with drought and other abiotic stresses [55], and the Gln/Glu and Asn/Asp ratios customarily used as markers of nitrogen cycling [56]. These ratios increased significantly in WT potato leaves under drought (4.3- and 1.9-fold, respectively), but not in *Stpfld252* plants (Figure S9). The results suggest that nitrogen mobilization was involved in this short-term water deficit condition, in agreement with the induction of genes involved in nitrogen uptake and assimilation observed in similar drought assays [27,56].

Finally, improvement of physiological and molecular stress responses by chloroplast Fld presence resulted in increased tuber yield under long-term non-lethal water restriction (Figure 9). While most drought- and Fld-dependent effects occurred and were monitored in leaves, it is likely that preservation of photosynthesis and other central metabolic pathways (e.g., glycolysis and starch synthesis) under water limitation favored production and transport of photosynthates from source to sink tissues. Further research will be necessary to identify the mechanisms by which the better biochemical performance of *Stpfld* leaves translated into improved tuber yield, and to evaluate the quality of the resulting tubers in terms of nutritional value and organoleptic properties.

Taken together, our results indicate that the Fld technology constitutes a remarkable tool to improve potato production under less-than-optimal conditions. Field trials are required to properly evaluate this possibility and its agronomic relevance.

## 4. Materials and Methods

### 4.1. Preparation and Growth of Potato Plants Expressing Cyanobacterial Fld

Construction of the pCAMBIA2200 vector encoding plastid-targeted Fld from *Anabaena* PCC7119 has been described elsewhere [15]. Briefly, a DNA sequence encoding the chloroplast transit peptide of pea ferredoxin-NADP^+^ reductase was fused in-frame to the 5′-end of the *fld* gene and placed under control of the cauliflower mosaic virus (CaMV) 35S promoter to allow for constitutive expression and plastid targeting in transformed plants (Figure S1a). Potato plants (*S. tuberosum* cv Solara) were transformed by agroinfiltration [23] to generate *Stpfld* lines. The presence of Fld in cleared leaf extracts was evaluated by SDS-PAGE and immunoblot detection with specific antisera [42]. Plants were propagated in vitro [23], transferred to soil, and grown at 400 μmol photons m^−2^ s^−1^, 16-h photoperiod, 25/22 °C and 80% relative humidity (growth chamber conditions).

For short-term drought assays, tuber slices of similar sizes from the various lines were planted in 3-L soil pots. Plants were watered to 100% FiC for 30 days and then subjected to drought by water withdrawal under growth chamber conditions.

For long-term drought treatments, tuber slices were planted in 15-L soil pots, and watered to 100% FiC for the first 30 days. By that time, plants were beginning to set tubers. Watering was interrupted until the soil reached 40% FiC and watered again to 70% FiC. This procedure was repeated until plants were harvested, 120 days post germination (Figure S7). RWC were determined in young fully expanded leaves from the fifth node according to Boguszewska et al. [57].

### 4.2. Photosynthetic Measurements

Chlorophyll a fluorescence determinations were performed using a MultispeQ-Beta device controlled by the PhotosynQ platform software [58]. Measurements were carried out at midday on leaves from the fifth node of 5 independent plants per line, and photosynthetic parameters were calculated according to Baker [24].

### 4.3. In Situ Detection of Reactive Oxygen Species

ROS cellular localization was determined by CLSM in an Eclipse TE–2000–E2 microscope (Nikon). Discs (110 mm^2^ in diameter) were collected from leaves belonging to the fifth node of 5 different plants per line and infiltrated with the ROS-sensitive fluorescent probe DCFDA as described by Mayta et al. [59]. Imaging was performed by scanning 5 optical slices (∼1 µm) of the palisade parenchyma immediately below the epidermis. Fluorescence intensities were estimated with the Fiji software [60], using z-projections of the different stacks.

### 4.4. RNA Isolation, cDNA Synthesis and Microarray Hybridization

For microarray analysis, 30-days old WT and *Stpfld252* plants were subjected to short-term drought treatment as described above. Young fully expanded leaves belonging to the fifth node were collected after three days of water withdrawal in the treated group. Leaf material from 10 different plants of each genotype and treatment were frozen in liquid nitrogen and ground with Mixer Mill MM400 (Retsch). Two pools of biological samples, each from two independent experiments per genotype and condition were used for the microarray analysis, resulting in two microarray experiments per genotype and condition. Leaf RNA was extracted according to Logemann et al. [61]. One microgram of RNA from each sample was treated with RQ1 DNase (Promega, Madison, WI, USA) according to the manufacturer’s instructions, and used as template to generate cDNA with M-MLV Reverse Transcriptase (Invitrogen, Carlsbad, CA, USA) and oligo (dT)12−18.

Gene expression profiles were assessed with the Potato Oligo Chip Initiative (POCI) microarray, a 60-mer oligo-based 4 × 44k Agilent microarray (AMADID: 015425), consisting of 42,034 60-mer probes [62]. Sample labeling and hybridization were performed as described in the one-color microarray-based gene expression analysis protocol including the one-color RNA spike-in Kit (v5.0.1, Agilent Technologies, Santa Clara, USA). Slides were scanned with an Agilent microarray scanner (G2505B) at high resolution. Data were extracted using feature extraction software (v9.5.3, Agilent, Santa Clara, CA, USA) by a standard protocol.

### 4.5. Microarray Data Analysis

Data processing and statistical analysis were carried out with the Bioconductor library limma [63]. Background correction and normalization were performed using the “normexp” and quantile methods, respectively. We only considered probes whose intensity was more than 10% above background on at least one genotype/treatment combination. An empirical Bayes method with moderated *t*-statistic was employed for determination of the genes with statistically significant changes, whereas Benjamini and Hochberg’s method was used to control FDR. DE genes were identified from pairwise comparisons when FDR < 0.05 and FC was >2 or <0.5. Pathway over-representation analyses between lines or treatment comparisons were performed with PageMan [64] using Fisher’s exact test with Bonferroni correction (FDR < 0.05). Mapman ontology was used for functional annotation [65] employing a mapping file updated in May 2018 (stu_Agilent_4 × 44k_2018-05-25_mapping.txt) from the GoMapMan website resource (www.gomapman.org, National Institute of Biology and Jožef Stefan Institute, Slovenia, [30]).

Based on the results of the multiple comparison test described above, genes were defined as induced (FC > 2 and FDR < 0.05), repressed (FC < 0.5 and FDR < 0.05) or unaffected in each of the four pairwise comparison combinations (*Stpfld252* vs. WT under control conditions, *Stpfld252* vs. WT under drought, drought vs. control in *Stpfld252*, drought vs. control in WT). Genes sharing the same results in the four pairwise comparisons were grouped into the same cluster and the corresponding graphs were plotted with the R library ggplot2 [66]. Pathway over-representation in each cluster was determined as described above.

### 4.6. Validation of DE Genes by qRT-PCR

Leaf samples for qRT-PCR analysis were obtained from independent experiments using the same drought treatment and plant ages as in the microarray assays. Total RNA was extracted from leaf tissue using TriPure (Sigma-Aldrich, St. Louis, USA) according to the manufacturer’s instructions, and reverse-transcribed with M-MLV (Invitrogen, Carlsbad, USA) as indicated before. The qRT-PCR reactions were carried out in a Master cycler Rep realplex4 thermocycler (Eppendorf) using Platinum Taq DNA polymerase (Invitrogen, Carlsbad, USA) and SYBR Green I (Roche) under the following conditions: 95 °C for 2 min and then 40 cycles of 95 °C for 15 s, 55 °C for 30 s, and 72 °C for 40 s. The relative abundance of transcripts was estimated with the ΔΔCt method [67], and normalized to the gene encoding elongation factor-1α (Genbank accession AB061263.1). Primers are listed in Table S8. Each qRT-PCR reaction set included 5–6 biological and 2 technical replicates, and water used as a negative no-template control instead of cDNA.

### 4.7. Metabolic Profiling

Sugars and amino acids were determined essentially as described by Ghaffari et al. [68]. Numerical analysis and quantification of individual compounds were carried out using the Empower Pro software (Waters, Milford, MA, USA) and authentic standards, respectively.

### 4.8. Availability of Supporting Data

Data reported in this publication have been deposited in NCBI’s Gene Expression Omnibus and are accessible through GEO Series accession number GSE149503 [69] (https://www.ncbi.nlm.nih.gov/geo/query/acc.cgi?acc=GSE149503).

### 4.9. Statistical Analyses

Determinations of photosynthetic activity, RWC, fluorescence intensity, qRT-PCR and metabolite levels were analyzed using one-way ANOVA and Tukey’s Multiple Comparison Test, whereas measurements of FW, water potential and tuber yield were analyzed with the non-parametric Mann–Whitney test. Statistical significance was specified in each experiment.

## Supplementary Materials

The following are available online at https://www.mdpi.com/1422-0067/21/19/7199/s1, Figure S1: Fld expression in leaves of transgenic potato plants. Figure S2: Relative water content during short-term drought. Figure S3: Low-populated clusters not shown in the main text Figures. Figure S4: Clusters formed by genes induced or repressed by drought independently of genotype. Figure S5: Comparison of expression patterns of selected DE genes as determined by microarray analysis (dark circles) and qRT-PCR (open circles). Figure S6: Amino acid profiling in leaves from WT and Fld-expressing potato plants. Figure S7: Long-term drought protocol. Figure S8: Comparison of drought-responsive DE genes in WT and Fld-expressing potato plants. Figure S9: Drought modified the ratios of amino acids related to nitrogen mobilization in WT but not *Stpfld* plants. Table S1: List of potato genes with altered expression patterns in response to Fld (Figure 3). Table S2: List of potato genes with altered expression patterns in response to drought in WT and Fld-expressing leaves (Figure 4). Table S3: List of potato genes belonging to clusters formed by drought-induced or repressed genes independently of genotype (Figure S4). Table S4: List of potato genes belonging to clusters formed by drought responses abolished or ameliorated by chloroplast Fld (Figure 5). Table S5: List of potato genes regulated by drought and Fld in opposite directions (Figure 6). Table S6: List of potato genes belonging to clusters formed by drought-responsive genes which were already primed by Fld in the absence of stress (Figure 7). Table S7: List of potato redox-associated genes differentially expressed in response to drought and/or Fld expression. Table S8: Primer sets used for qRT-PCR determinations.

## Abbreviations

ROS	reactive oxygen species
Fld	flavodoxin
Fd	ferredoxin
PETC	photosynthetic electron transport chain
WT	wild-type
SDS-PAGE	sodium dodecyl sulfate-polyacrylamide gel electrophoresis
RWC	relative water contents
PS	photosystem
NPQt	non-photochemical quenching of chlorophyll fluorescence
CLSM	confocal laser scanning microscopy
DCFDA	2′,7′-dichlorodihydrofluorescein diacetate
DE	differentially expressed
FC	fold-change
FDR	false discovery rate
ABA	abscisic acid
GABA	and γ-aminobutyrate
MIPS	myo-inositol-1-phosphate synthase
qRT-PCR	quantitative reverse-transcription
P5C	pyrroline-5-carboxylate
FiC	field capacity
CaMV	cauliflower mosaic virus
