# Supplementary figures and images for: Transcriptional and Metabolic Profiling of Potato Plants Expressing a Plastid-Targeted Electron Shuttle Reveal Modulation of Genes Associated to Drought Tolerance by Chloroplast Redox Poise
Source: Int J Mol Sci. 2020 Sep 29;21(19):7199. doi: 10.3390/ijms21197199 (PMC7582712; doi:10.3390/ijms21197199)

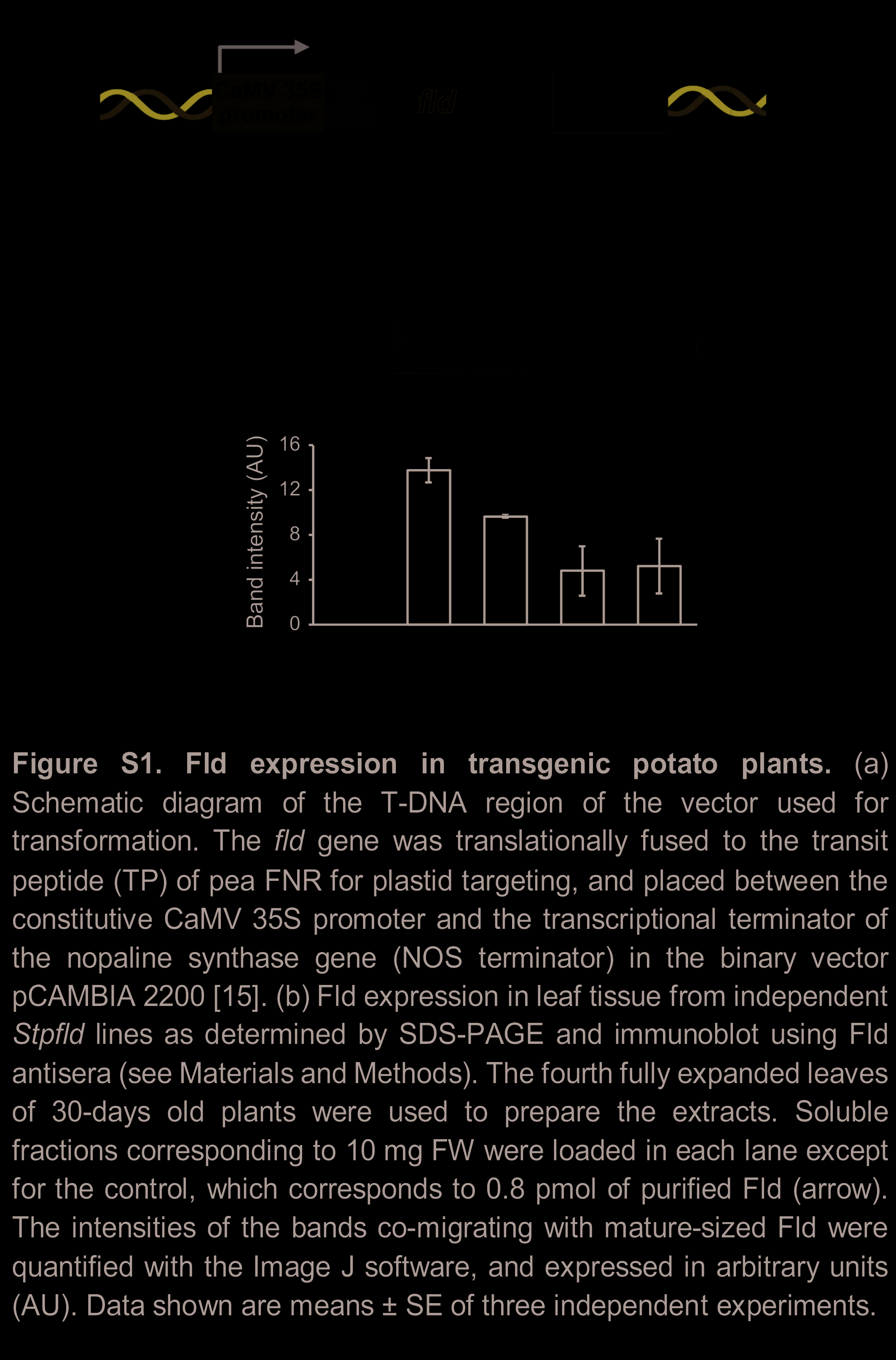

Supplement: Supplementary file 1 [file ijms-21-07199-s001.zip › Figure_S1.tif]

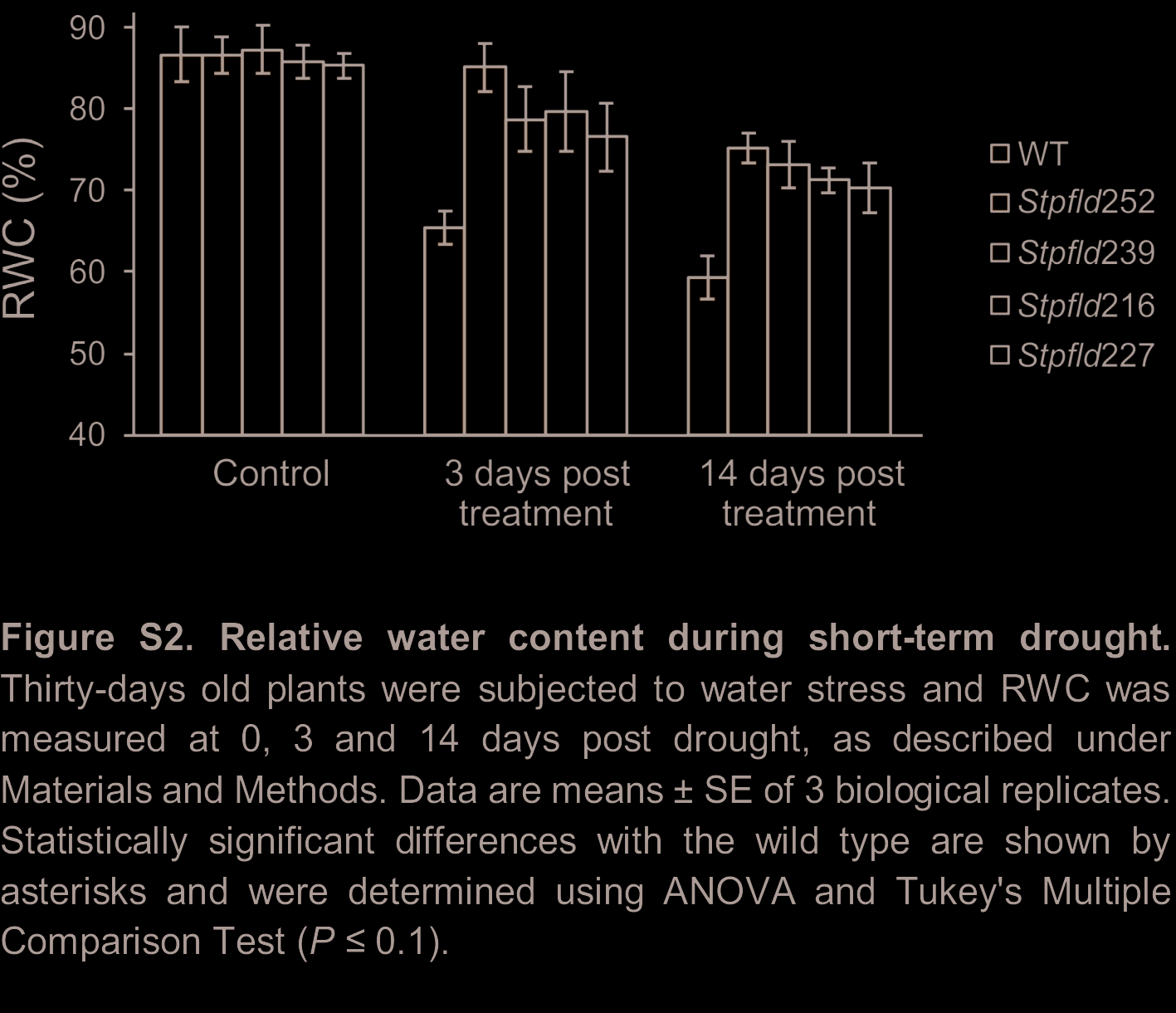

Supplement: Supplementary file 1 [file ijms-21-07199-s001.zip › Figure_S2.tif]

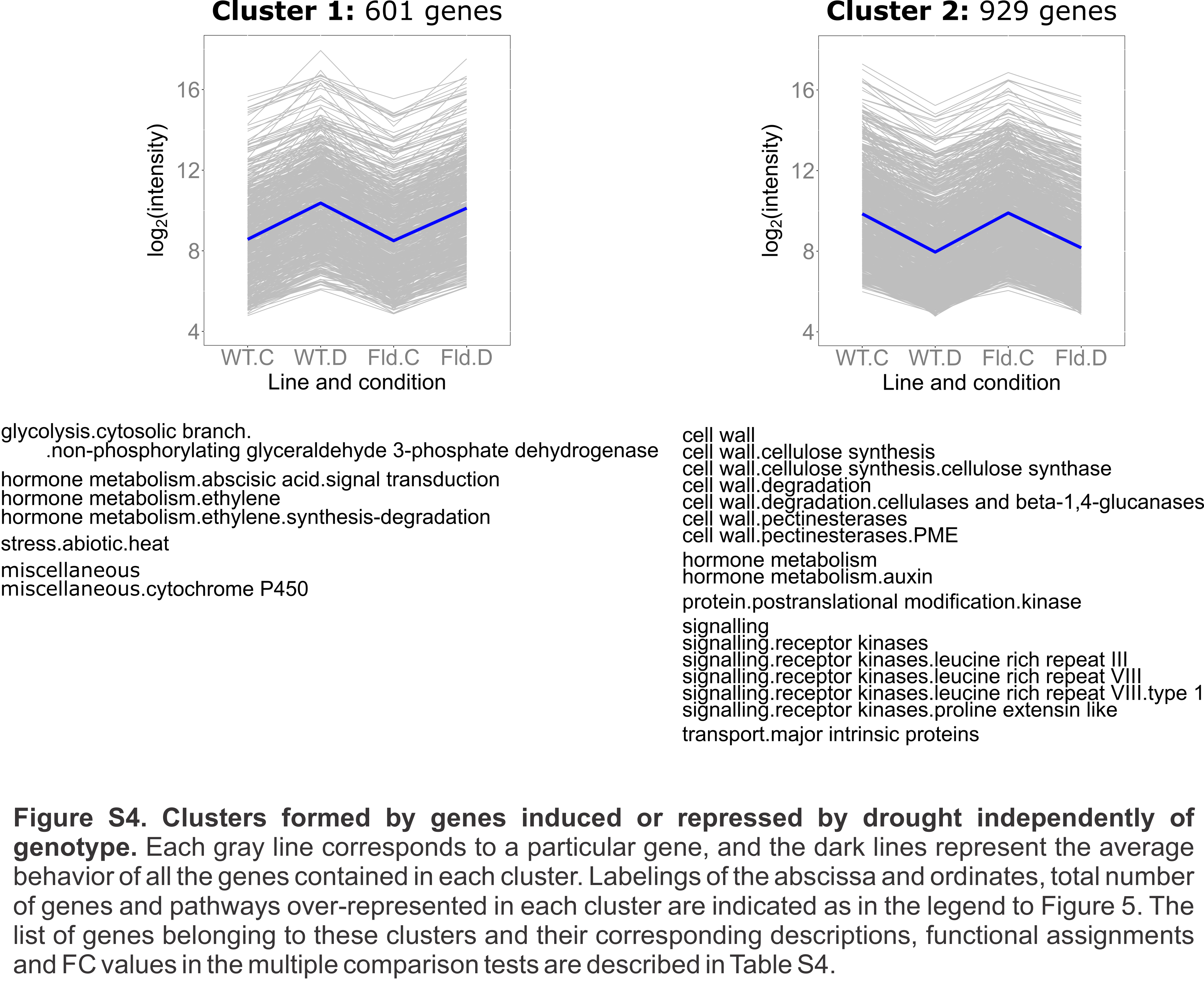

Supplement: Supplementary file 1 [file ijms-21-07199-s001.zip › Figure_S4.tiff]

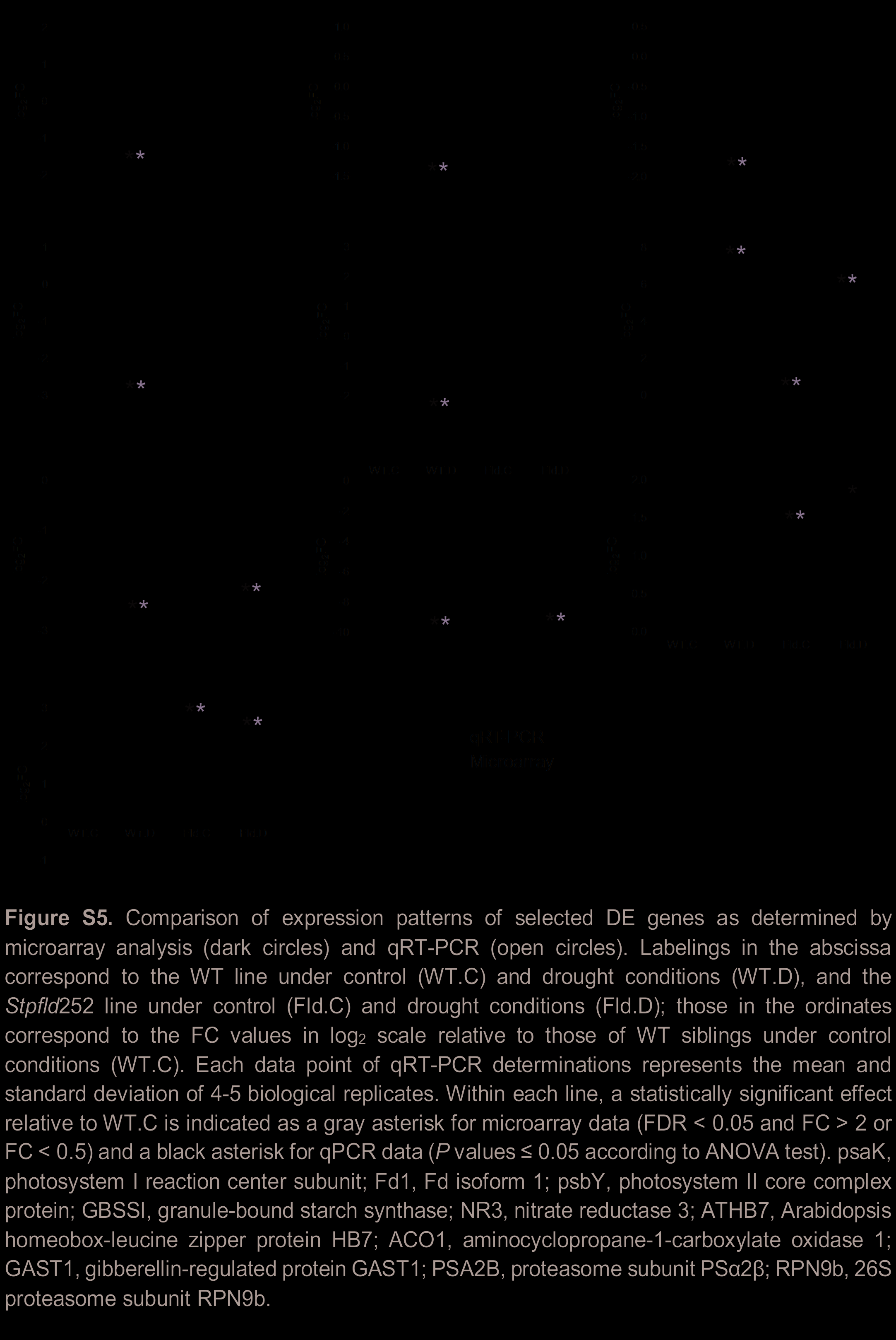

Supplement: Supplementary file 1 [file ijms-21-07199-s001.zip › Figure_S5.tif]

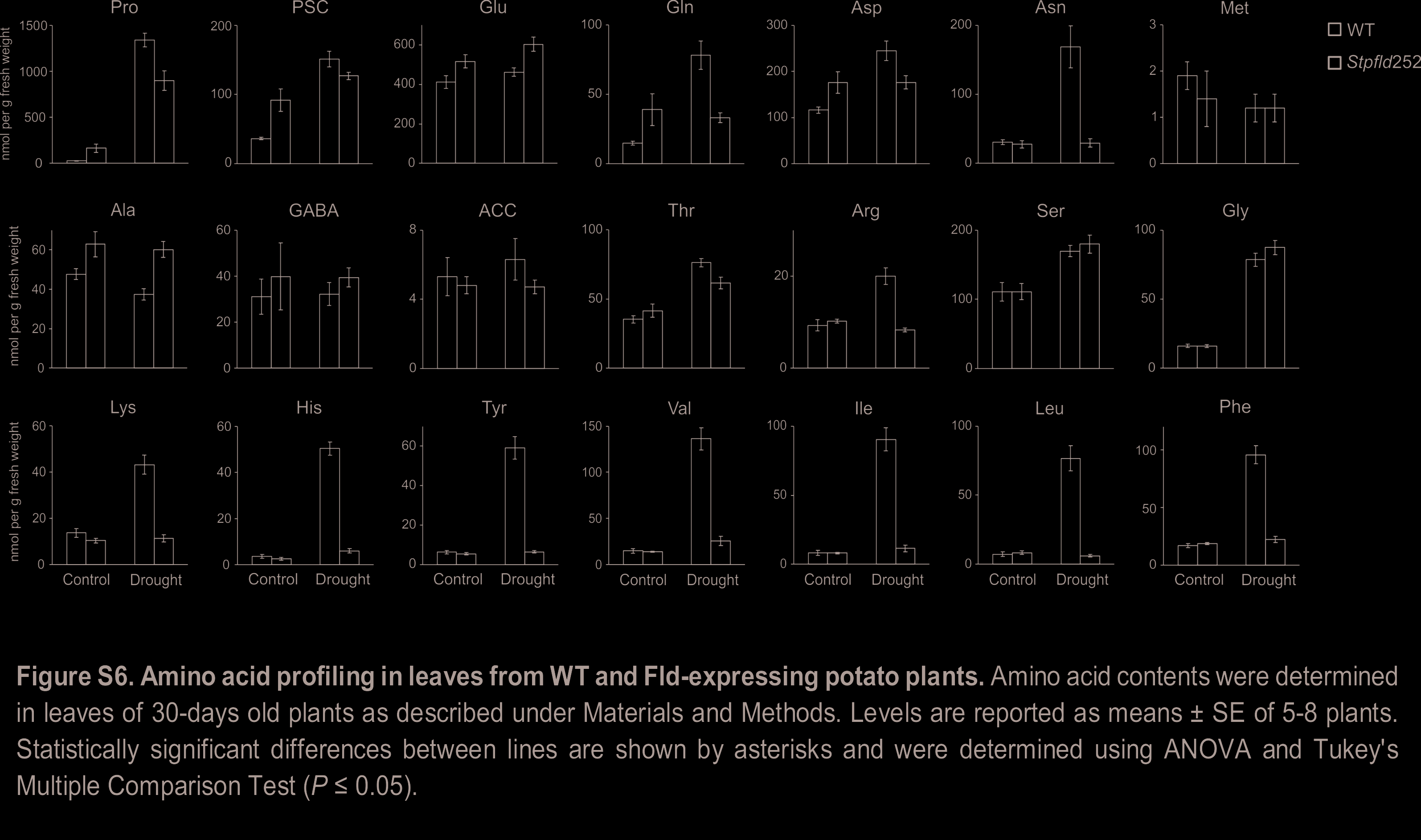

Supplement: Supplementary file 1 [file ijms-21-07199-s001.zip › Figure_S6.tif]

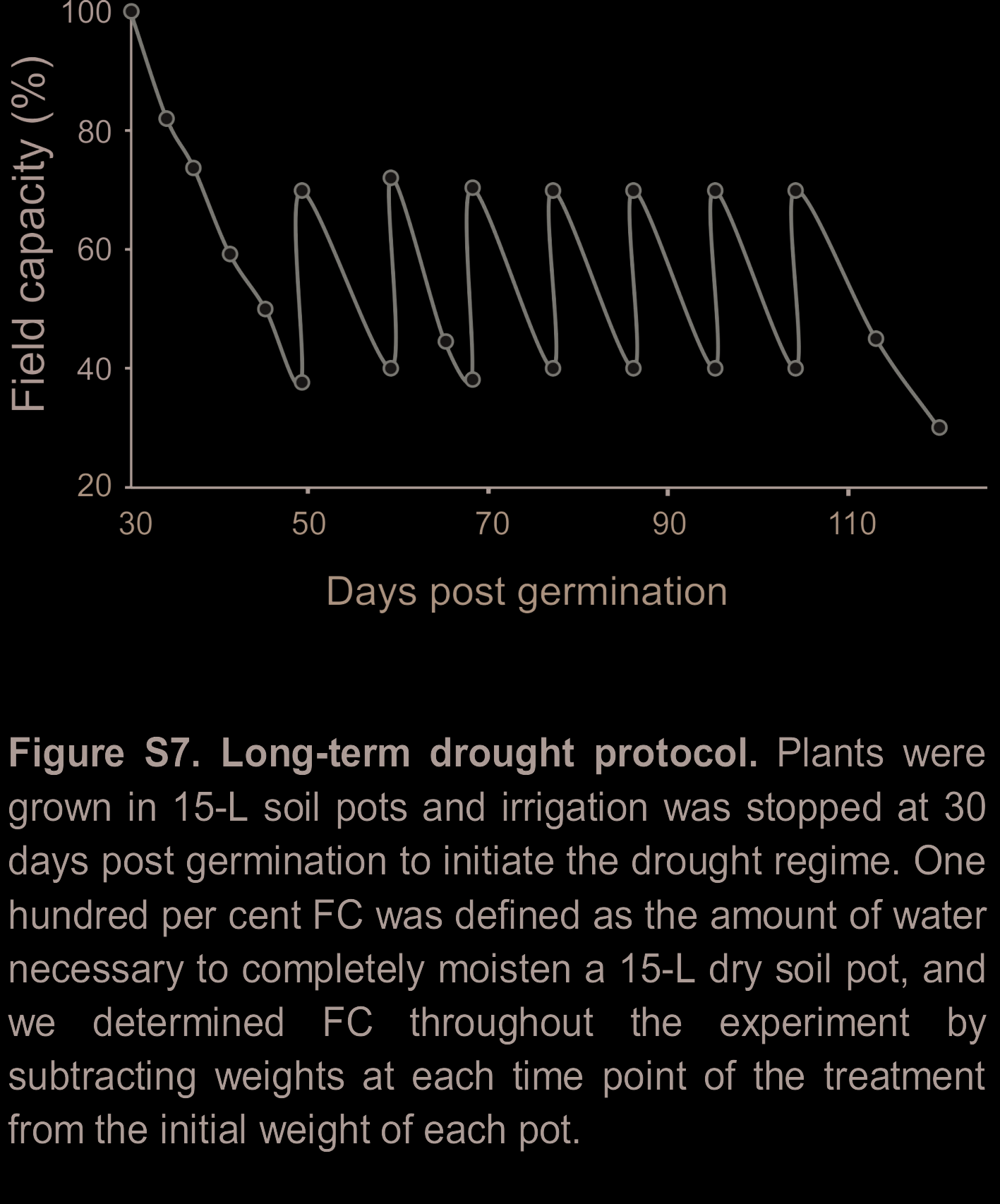

Supplement: Supplementary file 1 [file ijms-21-07199-s001.zip › Figure_S7.tif]

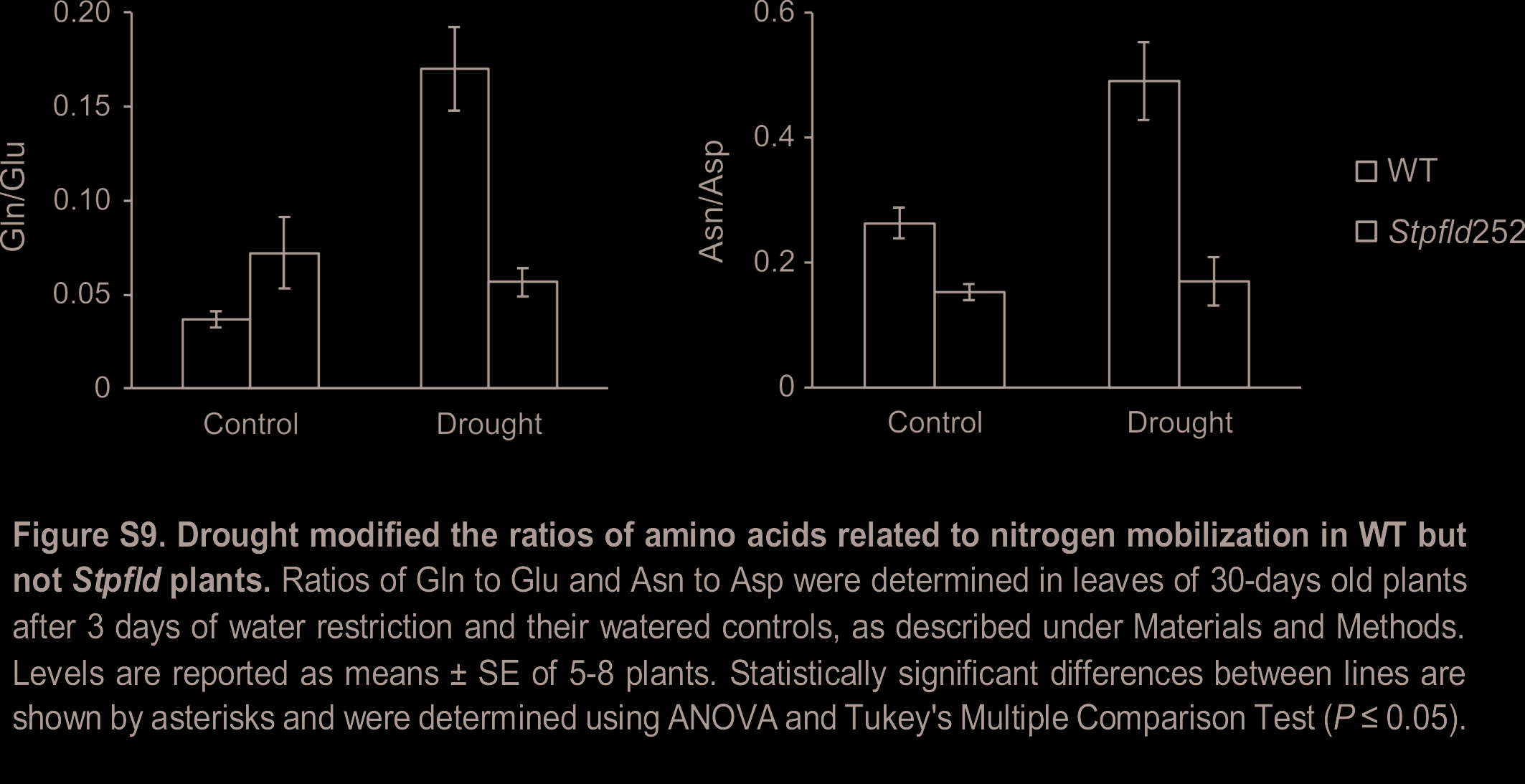

Supplement: Supplementary file 1 [file ijms-21-07199-s001.zip › Figure_S9.tif]
